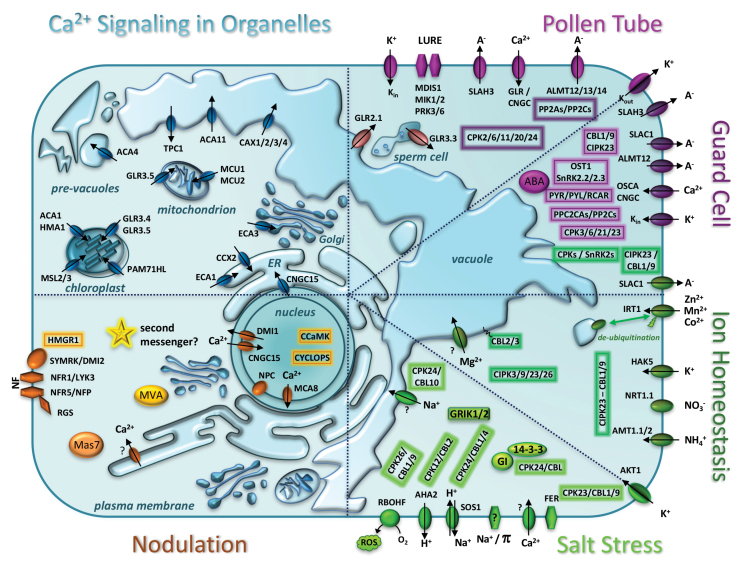# ‘Calcium is life’

**DOI:** 10.1093/jxb/ery279

**Published:** 2018-07-30

**Authors:** José A Feijó, Michael M Wudick

**Affiliations:** University of Maryland Department of Cell Biology and Molecular Genetics, College Park, MD, USA

**Keywords:** Ca^2+^-binding protein, Ca^2+^ channel, Ca^2+^ signaling, calcium, calmodulin, guard cells, mitochondria, nodulation, pollen tubes, stress tolerance

**Ca^2+^ signaling is critically important for cell and developmental biology. Despite long-standing issues still holding back the field, an increasingly large repertoire of genes and mechanisms has now been described. The unanticipated complexity revealed by genomics is giving way to a renaissance in our understanding, and the characterization of novel molecular mechanisms. The reviews in this special issue bring together research focused on specific structures, including mitochondria, pollen tubes and guard cells, as well as on the processes of ion homeostasis and salt stress tolerance, and nodulation.**

Back in 1995, a British scientist was driving past a research institute in the south of France when his attention was caught by an unusual road sign: ‘Le Calcium C’est La Vie’. A bizarre sight for any driver, a picture of it swiftly moved into the talks given by Anthony Trewavas, a leading Ca^2+^ signaling researcher, to signify the importance and relevance of calcium (Ca^2+^) as the most versatile signaling second messenger, involved in practically all aspects of cell and developmental biology from egg activation to cell apoptosis. Eventually the French pronouncement made it into the title of an essay about the nature and mechanisms behind Ca^2+^ waves in plants (Trewavas, 1999). The present special issue takes on the symbolic urgency of this road sign to highlight the centrality of Ca^2+^ signaling in practically every scenario that can be classed as ‘experimental botany’.

Key considerations for the reviews are novelty and the simultaneous need to address long-standing issues still holding back the field. For example, we lack fundamental knowledge on the apparent absence of any ligand-operated Ca^2+^ storage system, we are only just beginning to reveal the molecular identity of Ca^2+^ channels, and strong disagreement still reigns about Ca^2+^ channel gating and regulation. Despite this, an increasingly large repertoire of genes and mechanisms has been described in recent years, and there is a growing body of researchers contributing breakthroughs on many fronts, from the identification of *bona fide* Ca^2+^ channels in plants to the definition of putative Ca^2+^-signaling networks.

## Gene discovery, unanticipated complexity

Back in ‘Le Calcium C’est La Vie’ days, the feeling of excitement was similar. A number of Ca^2+^-binding proteins, putative transducers of the basic Ca^2+^ signals, were discovered by a combination of biochemistry and the first genetic screens, which were designed for the most essential aspects of plant biology. These revealed gene/protein families specific to plants, like the Calcium Dependent Protein Kinases (CPKs or CDPKs) and the CBL–CIPK (Calcineurin B-like protein and CBL-Interacting Protein Kinase) pairs. Some ‘usual suspects’, such as animal homologs of calmodulin and the CMLs (Calmodulin-Like proteins) were also confirmed as playing important roles (reviews in Harper *et al.*, 2004; Hepler, 2005). The field went ahead quickly on the basis of what looked like the roadmap for a true Ca^2+^ signature and signaling paradigm in plants, as was occurring in the animal field.

The advent of genomics, and the consequent reverse genetics approaches, brought tremendous speed to the gene discovery process, but rather than confirming a paradigm this revealed a great deal of unanticipated complexity, with members of most Ca^2+^-signaling protein families running into the dozens. Genomics also brought about a need to revise many pharmacological approaches due to the absence of homologs to the mammalian genes in light of which those assays were designed and interpreted. And there was the conundrum of what were the Ca^2+^ channels in plants, as no obvious family emerged from the Arabidopsis genome and multiple forward genetics screens over a decade or so failed to bring consensus about their genetic identity.

## Plants do it differently

There is now something of a renaissance in our understanding of many of these issues: there is some agreement about at least five families of Ca^2+^-permeable channels (Swarbreck *et al.*, 2013) and the involvement of differently coded Ca^2+^ signaling in various aspects of plant physiology seems beyond doubt (Dodd *et al.*, 2010; Edel and Kudla, 2015). A recent analysis of the evolutionary trends of Ca^2+^ signaling in plants (Edel *et al.*, 2017) focused on the fact that, when compared to animals, the available repertoire of genes coding for Ca^2+^-influx mechanisms in plants is reduced, and therefore the available machinery must shoulder a greater burden in terms of fulfilling the same signaling functions. The authors elaborate that this limitation on channel diversity is compensated by larger and more-diverse families of Ca^2+^-binding signaling proteins capable of contributing to the amplification and integration of the primary Ca^2+^ signals.

These are provocative conclusions that may be falsified if new families of channels are found, but suggest, as perhaps the most reasonable explanation for present findings, that plants ‘do it differently’. So although the animal paradigms served us well in searching for conservation of function, the time is ripe to assume that (i) even when the same molecular mechanisms are present, they may result from convergent evolution with adaptation to the very different contexts of plant physiology, and thus (ii) the same function may be achieved through different associations and regulatory mechanisms. Box 1 brings together the key elements of the novel molecular mechanisms described in the reviews in this special issue.

## Channels and stores

Of all the gene families documented as coding for Ca^2+^-permeable channels, the ones for which there are more data available are the Glutamate Receptor-Like (GLRs) and the Cyclic Nucleotide Gated channels (CNGCs).

GLRs made it to center stage directly from their genomic identification during the assembly of the Arabidopsis genome (Lam *et al.*, 1998). This is not surprising as there was little expectation of their existence in organisms without an organized nervous system. In Arabidopsis, the family has twenty genes divided into three clades and high functional redundancy, making this family more numerous than its homolog in our own human nervous system. A decade of primary screens allowed some advances in defining their physiological roles (reviews in Davenport, 2002; Konrad *et al.*, 2011; Forde and Roberts, 2014). Multiple functions have been attributed to GLRs, but the field was shaken by the demonstration that they may be involved in the conductance of long-range electrical signaling in response both to herbivore (Mousavi *et al.*, 2013) and aphid (Vincent *et al.*, 2017) feeding. Wudick *et al.* (2018) take a different perspective, and rather focus on the point that given the current uncertainties on regulation by oligomerization, ligand gating, ion specificity and association with other proteins, data from this kind of screening will always be difficult to interpret in terms of channel function. Further structural and evolutionary arguments are raised to make the case that elucidation of the molecular properties of these channels is needed for full understanding of their biological function, as GLRs stand as a good example of the limitations inherent to strictly translating mammalian knowledge of function and regulation.

Equally with 20 gene copies, but contrary to GLRs, some single mutant CNGCs seem highly unique in their phenotypes. CNGC18 was one of the first to be characterized (Frietsch *et al.*, 2007), with its single mutation resulting in an extremely strong pollen tube/reproductive phenotype. Other members show similarly strong phenotypes from single mutations, which is remarkable given the multitude of members, for which one would expect a high degree of redundancy. Another puzzling fact is our lack of knowledge on the pathways for synthesis and degradation of any type of cyclic nucleotides in plants. Yet, of relevance, CNGC15 was found to be essential for the generation of Ca^2+^ signatures in the nuclei of *Medicago* root cells during *Rhizobium* infection (Charpentier *et al.*, 2016). This was the last and most elusive member of the cascade of proteins involved in the propagation of Ca^2+^ signals triggered by Nod factors along the root hair, where the nodulation transcriptional program is triggered in the nucleus upon a specific number of Ca^2+^ elevations.

Nuclear Ca^2+^ oscillations have been known for a long time (e.g. Pauly *et al.*, 2000), and in this issue Charpentier (2018) contextualizes the nodulation signal based on all the reported nuclear Ca^2+^ signaling phenomena described in plants. The nodulation case study is then used as a template to discuss the origin of nuclear signals in diverse contexts and the mechanisms of downstream transcriptional regulation, clearly suggesting a role for the nuclear envelope as an important Ca^2+^ store capable of generating specific transcriptional triggering signatures, namely through CNGCs.

The whole issue of Ca^2+^ stores is taken to a new level in the review by Costa *et al.* (2018). These authors bring together what we know about the main intracellular Ca^2+^ stores: the vacuole, endoplasmic reticulum, Golgi, peroxisomes, apoplast, and the double membrane organelles, the mitochondria and plastids. Special attention is given to the latter two, as the authors have been at the forefront of the molecular characterization of the channels involved in Ca^2+^ transport from mitochondria and plastids. Some GLRs (3.4 and 3.5) have distinct peptide signals that target these organelles, and the authors were pioneers in showing that to be the case and so implicating them in Ca^2+^ homeostasis (Teardo *et al.*, 2015).

More profoundly the team has been at the forefront in characterizing the mitochondrial channel uniporter (MCU) in plants (Teardo *et al.*, 2017). These transporters were long sought, their existence implied by a number of mitochondrial Ca^2+^ pathologies, and first demonstrated by Rizzuto’s team (De Stefani *et al.*, 2011). Given their importance for cytosolic Ca^2+^ homeostasis in mammalian cells, their discovery in plants bears promise of equally relevant functions. Besides thorough coverage of the molecular mechanisms operating in all these organelles and how they make functional Ca^2+^ stores, Costa *et al.* (2018) also offer arguably the most extensive and comprehensive published account of Ca^2+^-imaging sensors (and methods for each), with critical comparisons from the leading group in the world in this area.

## Codes, networks and stress

The hallmark of Ca^2+^ signaling is the formation of unique spatial and temporal patterns of cytosolic concentration changes that carry specific information. These are collectively known as Ca^2+^ signatures, and include oscillations, elevations, standing waves and, more rarely, standing gradients. The holy grail of the field is to know exactly how these patterns encode information, and how specific proteins that bind Ca^2+^ with different affinities and kinetics are able to decode them, resulting in specific modifications (e.g. phosphorylation/de-phosphorylation) of other downstream proteins. Konrad *et al.* (2018) focus on two systems with Ca^2+^ oscillation either on a standing gradient (the pollen tube) or spatially distributed (guard cells/stomata) to infer common patterns and different properties that could help explain the network of interactions, feedback loops and pattern-generation mechanisms. Both systems have been extensively used for Ca^2+^-signaling research, but the meaning of their Ca^2+^ signatures remains elusive.

Pollen tubes possess arguably the most robust and conspicuous standing Ca^2+^ gradients of any cell at their growing tip, and when germinated *in vitro* display oscillations in many species. However, this is not always the case, and there are no sound data showing that they exist *in vivo* (Damineli *et al.*, 2017). Guard cells, on the other hand, stand together with nodulation as one of the two examples where a certain number of elevations have been shown and suggested to have a physiological function, in this case the closure of the stomata (Allen *et al.*, 2000).

Konrad *et al.* (2018) cover all the known families of Ca^2+^-binding proteins, but with a bias for the CPKs, the area in which the authors have contributed most significantly. Some original data are presented on Ca^2+^ dynamics during fast stomata closure. A comparison between the ionic regulation of these two systems has been published before (Michard *et al.*, 2017), the originality here being the greater molecular detail and definition of a set of behaviors collectively designated ‘signalosomes’. Comparison of the signalosomes is used to establish correlations between genetics, spatial and temporal patterns, and biochemistry; these are then built into a comparative model that suggests that pollen tubes and stomata seem to operate through the same sort of functional units to generate the two macroscopic outputs of these cells, growth and closure, respectively. These kind of parallels are useful as a narrative and to inspire experiments to test the underlying hypotheses in terms of temporal delays, which can be measured with ever-increasing efficiency as new probes become available (see Costa *et al.*, 2018, for probe choice) and as the group has recently shown (Guttermuth *et al.*, 2018).

Concluding the issue, Manishankar *et al.* (2018) review the very competitive field of Ca^2+^ signaling during salt stress. Salt stress is simultaneously one of the most profound abiotic stress problems and one of the most successful stories in which non-biased genetic screens have led to the discovery of completely unsuspected and original molecular mechanisms in plants. The first such mutants were of the class SOS (salt overly sensitive; Liu and Zhu, 1997) and gave rise to one of the most dynamic fronts of research on Ca^2+^ decoding, involving the CBL–CIPK sensor (Kudla *et al.*, 1999). This sensor arguably constituted the first identified pathway for ion homeostasis in plants and is triggered by Ca^2+^ binding giving rise to numerous and eloquent reviews on the subject (e.g. Edel and Kudla, 2015).

The huge number of possible combinations between the members of the two families (10 CBLs×26 CIPKs in Arabidopsis) constitutes a formidable challenge such that all combinations are tested under specific screens. Nevertheless, the prospect that some of these combinations might bear the right kinetics and affinities to make them ‘the’ specific sensor for a certain Ca^2+^ signature is tantalizing. Manishankar *et al.* (2018) cover the abundant literature that relates to specific CBL–CIPKs as being associated with specific kinds of salt stress responses, namely for potassium, nitrogen molecules, magnesium, metals and anions, and argue that CBL–CIPKs have a ‘…coordinated role for Ca^2+^ signaling in plant nutrition’. As with the review by Konrad *et al.* (2018), the core of the system consists of the phosphorylation of specific ion channels that in return affect Ca^2+^ concentration, providing the feedback loop for Ca^2+^ binding to the kinase or kinase complex, respectively.

## Conclusion

The representation of novel molecular mechanisms provided in Box 1 highlights how much progress the Ca^2+^-signaling field is experiencing. In addition, it shows the fragmentation that has occurred into each specialist area, which calls for a more systems-oriented perspective to integrate these different parts. The reviews in this issue provide challenging perspectives on ways to reach this goal, but achieving it would lay the ground for the next steps where the formation of waves and the decoding of specific signatures still lack defined molecular mechanisms.

**Box 1. Ca^2+^ signaling in the plant cell**

Unified representation of Ca^2+^ signaling in the plant cell, with different types of organization color coded by quadrant of the ‘textbook’ diagram. Moving clockwise: (i) structures—mitochondria (Costa *et al.*, 2018), pollen tubes (Wudick *et al.*, 2018), and guard cells (Konrad *et al.*, 2018); (ii) processes —ion homeostasis and salt stress tolerance (Manishankar *et al.*, 2018), and nodulation (Charpentier, 2018).